# Crystal structure of tin(IV) chloride octa­hydrate

**DOI:** 10.1107/S1600536814024271

**Published:** 2014-11-12

**Authors:** Erik Hennings, Horst Schmidt, Wolfgang Voigt

**Affiliations:** a TU Bergakademie Freiberg, Institute of Inorganic Chemistry, Leipziger Strasse 29, D-09596 Freiberg, Germany

**Keywords:** crystals structure, low-temperature salt hydrates, tin(IV) salts

## Abstract

The title compound, [SnCl_4_(H_2_O)_2_]·6H_2_O, was crystallized according to the solid–liquid phase diagram at lower temperatures. It is built-up of SnCl_4_(H_2_O)_2_ octa­hedral units (point group symmetry 2) and lattice water mol­ecules. An intricate three-dimensional network of O—H⋯O and O—H⋯Cl hydrogen bonds between the complex molecules and the lattice water molecules is formed in the crystal structure.

## Abstract 2

The title compound was crystallized according to the solid–liquid phase diagram at lower temperatures. It is built-up of SnCl_4_(H_2_O)_2_ octa­hedral units and lattice water mol­ecules. An intricate three-dimensional network of O—H⋯O and O—H⋯Cl hydrogen bonds between the complex molecules and the lattice water molecules is formed in the crystal structure.

## Chemical context   

The inter­est in the stability of tin(IV) salts, especially at lower temperatures, has increased with the recent new determination of the redox potential in aqueous solutions, which is complicated by the presence of chlorido complexes (Gajda *et al.*, 2009 ▶). The phase diagram of tin(IV) chloride is not well investigated. Only some points in dilute solutions have been determined by Loomis (1897 ▶). For the existing hydrates (*R* = 8, 5, 4, 3 and 2), Meyerhoffer (1891 ▶) described the melting points and the existence fields. The crystal structures of the dihydrate (Semenov *et al.*, 2005 ▶), trihydrate (Genge *et al.*, 2004 ▶; Semenov *et al.*, 2005 ▶), tetra­hydrate (Genge *et al.*, 2004 ▶; Shihada *et al.*, 2004 ▶) and penta­hydrate (Barnes *et al.*, 1980 ▶; Shihada *et al.*, 2004 ▶) have been determined previously. For these salt hydrates, vibrational spectra are also available, classifying all hydrate spectra with point group *D*_4*h*_ symmetry (Brune & Zeil, 1962 ▶).

## Structural commentary   

**Figure 1:**
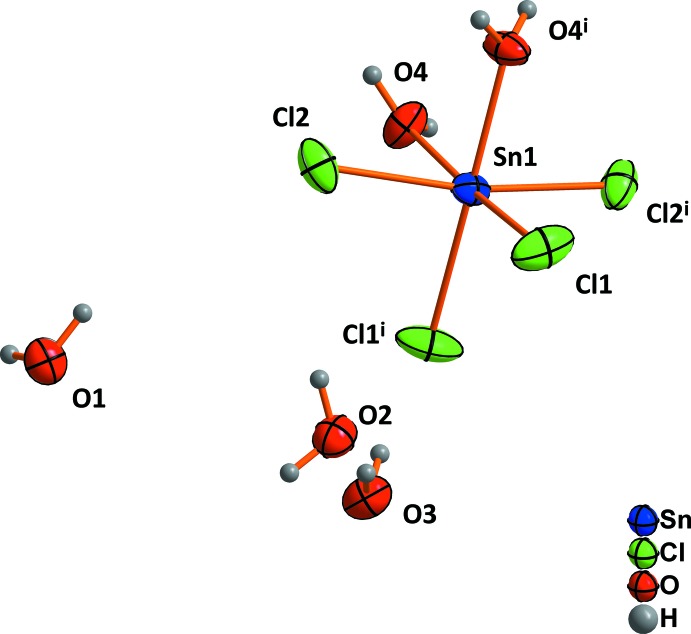
The building units in tin(IV) chloride octa­hydrate [symmetry code: (i) −*x*, *y*, −*z* + 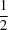]. Displacement ellipsoids are drawn at the 50% probability level.

**Figure 2:**
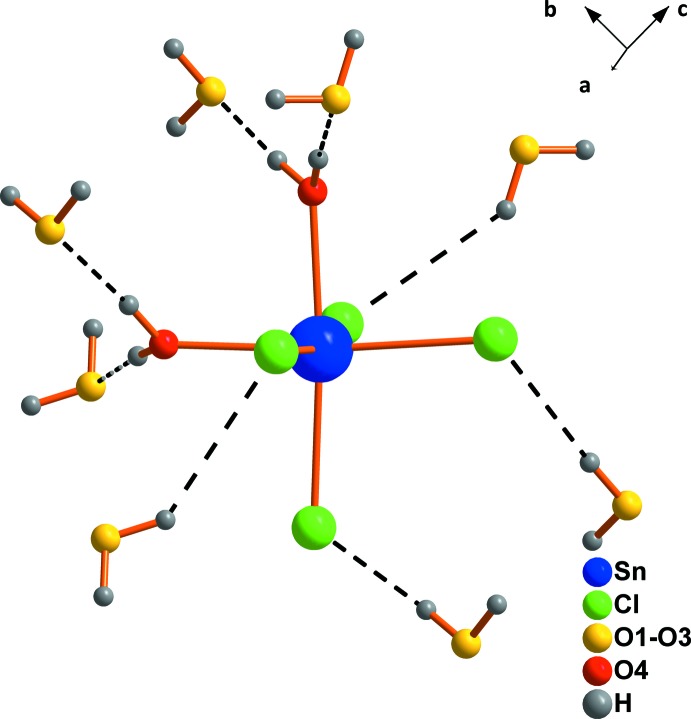
The coordination of tin(IV) in the second coordination shell of tin(IV) chloride octa­hydrate [symmetry code: (i) −*x*, *y*, −*z* + 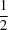]. Hydrogen bonds are shown as dashed lines.

The tin(IV) ion in tin(IV) chloride octa­hydrate is situated on a twofold rotation axis and is coordinated by four Cl atoms and two water mol­ecules in a *cis*-octahedral geometry (Fig. 1 ▶), as was observed before for the tetra- and penta­hydrate (Shihada *et al.*, 2004 ▶). In addition, three water mol­ecules (O1, O2 and O3) are located around the octa­hedra as non-coordinating water mol­ecules. Every water mol­ecule of the first coordination sphere is connected with two water mol­ecules of the second shell by hydrogen bonds. The chlorine atoms form only one hydrogen bond towards ‘free’ water mol­ecules of the second shell (Fig. 2 ▶).

## Supra­molecular features   

**Figure 3:**
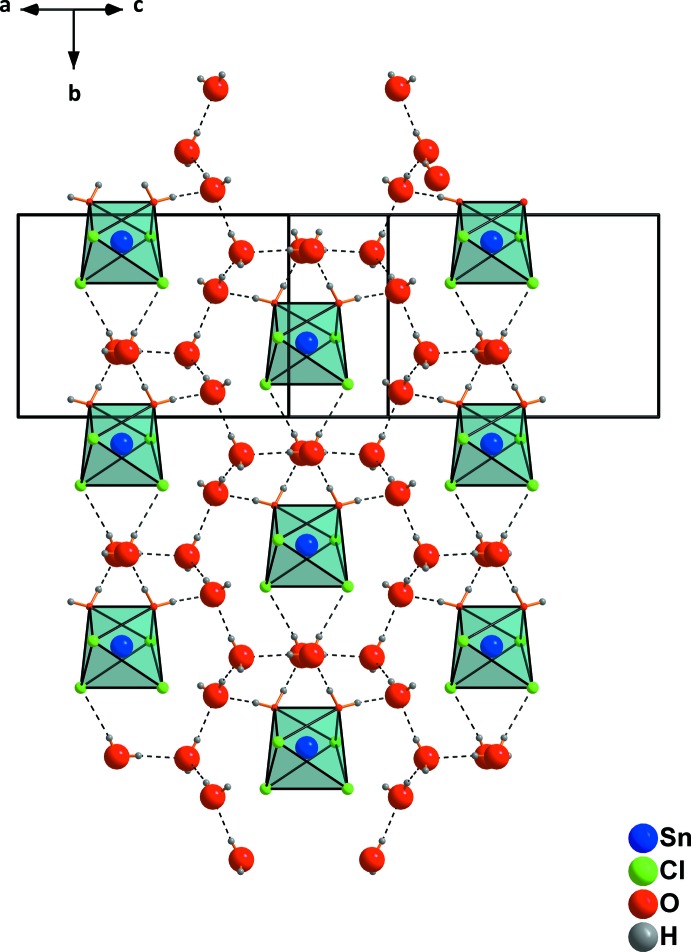
Formation of chains by water mol­ecules O1 and O2 (bold). Dashed lines indicate hydrogen bonds.

Having a larger view of the crystal structure in direction [001] (Fig. 3 ▶), it becomes obvious that these non-coordinating water mol­ecules form chains between the octa­hedrally coordinated tin(IV) ions. These water mol­ecules (O1 and O2) are connected *via* hydrogen bonds (Table 1 ▶) and the chains are oriented along the *b*-axis direction. Considering all types of hydrogen bonding, a three-dimensional network between the complex molecules and the lattice water molecules results.

## Database survey   

For crystal structure determination of other tin(IV) chloride hydrates, see: Shihada *et al.* (2004 ▶); Semenov *et al.* (2005 ▶); Genge *et al.* (2004 ▶); Barnes *et al.* (1980 ▶).

## Synthesis and crystallization   

Tin(IV) chloride octa­hydrate was crystallized from an aqueous solution of 53.39 wt% SnCl_4_ at 263 K after 2 d. For preparing this solution, tin(IV) chloride penta­hydrate (Acros Organics, 98%) was used. The content of Cl^−^ was analysed by titration with AgNO_3_. The crystals are stable in their saturated solution over a period of at least four weeks.

The samples were stored in a freezer or a cryostat at low temperatures. The crystals were separated and embedded in perfluorinated ether for X-ray diffraction analysis

## Refinement   

Crystal data, data collection and structure refinement details are summarized in Table 2 ▶. The H atoms were placed in the positions indicated by difference Fourier maps. Distance restraints were applied for the geometries of all water molecules, with O—H and H—H distance restraints of 0.84 (1) and 1.4 (1) Å, respectively.

## Supplementary Material

Crystal structure: contains datablock(s) I. DOI: 10.1107/S1600536814024271/br2243sup1.cif

Structure factors: contains datablock(s) I. DOI: 10.1107/S1600536814024271/br2243Isup2.hkl

CCDC reference: 1032661

Additional supporting information:  crystallographic information; 3D view; checkCIF report

## Figures and Tables

**Table 1:** Hydrogen-bond geometry (, )

*D*H*A*	*D*H	H*A*	*D* *A*	*D*H*A*
O1H1*B*O2^i^	0.84(1)	1.90(2)	2.729(3)	169(6)
O2H2*B*O3^ii^	0.84(1)	2.04(2)	2.825(3)	157(5)
O2H2*A*O1^iii^	0.84(1)	1.94(2)	2.762(3)	168(5)
O1H1*A*Cl3^iv^	0.84(1)	2.68(3)	3.389(2)	143(4)
O3H3*A*O2	0.84(1)	1.95(2)	2.763(3)	163(4)
O3H3*B*Cl1^v^	0.83(1)	2.43(1)	3.260(2)	173(4)
O4H4*B*O1^vi^	0.83(1)	1.77(1)	2.598(3)	176(4)
O4H4*A*O3^vii^	0.84(1)	1.80(1)	2.635(3)	176(4)

Symmetry codes: (i) 
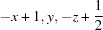
; (ii) 
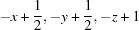
; (iii) 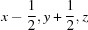; (iv) 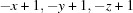; (v) 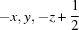; (vi) 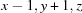; (vii) 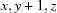.

**Table 2:** Experimental details

Crystal data	
Chemical formula	[SnCl_4_(H_2_O)_2_]6H_2_O
*M*_r_	404.62
Crystal system, space group	Monoclinic, *C*2/*c*
Temperature (K)	200
*a*, *b*, *c* ()	16.0224(15), 7.8530(8), 12.6766(12)
()	119.739(7)
*V* (^3^)	1384.9(2)
*Z*	4
Radiation type	Mo *K*
(mm^1^)	2.63
Crystal size (mm)	0.34 0.23 0.12

Data collection	
Diffractometer	Stoe IPDS 2T
Absorption correction	Integration (Coppens, 1970 ▶)
*T*_min_, *T*_max_	0.492, 0.731
No. of measured, independent and observed [*I* > 2(*I*)] reflections	13041, 1600, 1451
*R*_int_	0.030
(sin /)_max_ (^1^)	0.650

Refinement	
*R*[*F* ^2^ > 2(*F* ^2^)], *wR*(*F* ^2^), *S*	0.021, 0.049, 1.11
No. of reflections	1600
No. of parameters	92
No. of restraints	12
H-atom treatment	All H-atom parameters refined
_max_, _min_ (e ^3^)	1.01, 0.71

Computer programs: *X-AREA* and *X-RED* (Stoe Cie, 2009 ▶), *SHELXS97* and *SHELXL2012* (Sheldrick, 2008 ▶), *DIAMOND* (Brandenburg, 2006 ▶) and *publCIF* (Westrip, 2010 ▶).

## supplementary crystallographic information

### Crystal data

**Table:**

[SnCl_4_(H_2_O)_2_]·6H_2_O	*F*(000) = 792
*M**_r_* = 404.62	*D*_x_ = 1.941 Mg m^−^^3^
Monoclinic, *C*2/*c*	Mo *K*α radiation, λ = 0.71073 Å
*a* = 16.0224 (15) Å	Cell parameters from 13366 reflections
*b* = 7.8530 (8) Å	θ = 1.8–29.6°
*c* = 12.6766 (12) Å	µ = 2.63 mm^−^^1^
β = 119.739 (7)°	*T* = 200 K
*V* = 1384.9 (2) Å^3^	Plate, colourless
*Z* = 4	0.34 × 0.23 × 0.12 mm

### Data collection

**Table:**

Stoe IPDS 2T diffractometer	1600 independent reflections
Radiation source: fine-focus sealed tube	1451 reflections with *I* > 2σ(*I*)
Detector resolution: 6.67 pixels mm^-1^	*R*_int_ = 0.030
rotation method scans	θ_max_ = 27.5°, θ_min_ = 2.9°
Absorption correction: integration (Coppens, 1970)	*h* = −22→21
*T*_min_ = 0.492, *T*_max_ = 0.731	*k* = −10→10
13041 measured reflections	*l* = −17→17

### Refinement

**Table:**

Refinement on *F*^2^	12 restraints
Least-squares matrix: full	Hydrogen site location: difference Fourier map
*R*[*F*^2^ > 2σ(*F*^2^)] = 0.021	All H-atom parameters refined
*wR*(*F*^2^) = 0.049	*w* = 1/[σ^2^(*F*_o_^2^) + (0.0133*P*)^2^ + 4.9358*P*] where *P* = (*F*_o_^2^ + 2*F*_c_^2^)/3
*S* = 1.11	(Δ/σ)_max_ = 0.001
1600 reflections	Δρ_max_ = 1.01 e Å^−^^3^
92 parameters	Δρ_min_ = −0.71 e Å^−^^3^

### Special details

**Table:**

Geometry. All esds (except the esd in the dihedral angle between two l.s. planes) are estimated using the full covariance matrix. The cell esds are taken into account individually in the estimation of esds in distances, angles and torsion angles; correlations between esds in cell parameters are only used when they are defined by crystal symmetry. An approximate (isotropic) treatment of cell esds is used for estimating esds involving l.s. planes.

### Fractional atomic coordinates and isotropic or equivalent isotropic displacement parameters (Å^2^)

**Table:**

	*x*	*y*	*z*	*U*_iso_*/*U*_eq_	
Sn1	0.0000	0.86619 (3)	0.2500	0.02527 (8)	
Cl3	0.17022 (5)	0.89264 (10)	0.37662 (6)	0.04076 (17)	
O4	−0.00709 (13)	1.0623 (3)	0.35862 (18)	0.0342 (4)	
Cl1	0.01591 (7)	0.66227 (10)	0.12053 (8)	0.0504 (2)	
O1	0.83339 (14)	0.1224 (3)	0.35849 (18)	0.0341 (4)	
O2	0.24917 (15)	0.3119 (3)	0.34936 (19)	0.0377 (4)	
O3	0.11311 (15)	0.3211 (3)	0.4228 (2)	0.0380 (4)	
H4A	0.030 (2)	1.146 (3)	0.376 (3)	0.057 (11)*	
H4B	−0.0574 (14)	1.086 (4)	0.359 (3)	0.044 (9)*	
H3B	0.085 (3)	0.414 (3)	0.414 (4)	0.070 (13)*	
H3A	0.145 (3)	0.326 (6)	0.387 (4)	0.082 (15)*	
H1A	0.837 (3)	0.169 (5)	0.420 (2)	0.063 (12)*	
H2A	0.270 (4)	0.405 (3)	0.341 (5)	0.100 (18)*	
H2B	0.291 (3)	0.251 (5)	0.404 (3)	0.099 (18)*	
H1B	0.805 (4)	0.190 (6)	0.299 (4)	0.14 (2)*	

### Atomic displacement parameters (Å^2^)

**Table:**

	*U*^11^	*U*^22^	*U*^33^	*U*^12^	*U*^13^	*U*^23^
Sn1	0.03433 (13)	0.02255 (12)	0.02383 (12)	0.000	0.01815 (10)	0.000
Cl3	0.0320 (3)	0.0591 (4)	0.0315 (3)	0.0162 (3)	0.0161 (3)	0.0064 (3)
O4	0.0290 (9)	0.0348 (10)	0.0440 (10)	−0.0046 (8)	0.0220 (8)	−0.0141 (9)
Cl1	0.0809 (6)	0.0363 (4)	0.0615 (5)	−0.0147 (4)	0.0563 (5)	−0.0188 (3)
O1	0.0356 (10)	0.0410 (11)	0.0311 (9)	0.0046 (8)	0.0206 (8)	−0.0013 (8)
O2	0.0387 (11)	0.0412 (11)	0.0372 (10)	−0.0055 (9)	0.0218 (9)	−0.0020 (9)
O3	0.0369 (10)	0.0334 (10)	0.0508 (12)	−0.0016 (8)	0.0273 (10)	−0.0054 (9)

### Geometric parameters (Å, º)

**Table:**

Sn1—O4	2.1064 (18)	Sn1—Cl3	2.3906 (7)
Sn1—O4^i^	2.1064 (18)	Sn1—Cl1	2.3954 (7)
Sn1—Cl3^i^	2.3906 (7)	Sn1—Cl1^i^	2.3954 (7)
			
O4—Sn1—O4^i^	86.01 (12)	Cl3^i^—Sn1—Cl1	94.12 (3)
O4—Sn1—Cl3^i^	87.81 (6)	Cl3—Sn1—Cl1	92.55 (3)
O4^i^—Sn1—Cl3^i^	84.90 (5)	O4—Sn1—Cl1^i^	88.99 (6)
O4—Sn1—Cl3	84.90 (5)	O4^i^—Sn1—Cl1^i^	174.47 (6)
O4^i^—Sn1—Cl3	87.81 (6)	Cl3^i^—Sn1—Cl1^i^	92.55 (3)
Cl3^i^—Sn1—Cl3	170.03 (4)	Cl3—Sn1—Cl1^i^	94.12 (3)
O4—Sn1—Cl1	174.47 (6)	Cl1—Sn1—Cl1^i^	96.09 (4)
O4^i^—Sn1—Cl1	88.99 (6)		

Symmetry code: (i) −*x*, *y*, −*z*+1/2.

### Hydrogen-bond geometry (Å, º)

**Table:**

*D*—H···*A*	*D*—H	H···*A*	*D*···*A*	*D*—H···*A*
O1—H1*B*···O2^ii^	0.84 (1)	1.90 (2)	2.729 (3)	169 (6)
O2—H2*B*···O3^iii^	0.84 (1)	2.04 (2)	2.825 (3)	157 (5)
O2—H2*A*···O1^iv^	0.84 (1)	1.94 (2)	2.762 (3)	168 (5)
O1—H1*A*···Cl3^v^	0.84 (1)	2.68 (3)	3.389 (2)	143 (4)
O3—H3*A*···O2	0.84 (1)	1.95 (2)	2.763 (3)	163 (4)
O3—H3*B*···Cl1^i^	0.83 (1)	2.43 (1)	3.260 (2)	173 (4)
O4—H4*B*···O1^vi^	0.83 (1)	1.77 (1)	2.598 (3)	176 (4)
O4—H4*A*···O3^vii^	0.84 (1)	1.80 (1)	2.635 (3)	176 (4)

Symmetry codes: (i) −*x*, *y*, −*z*+1/2; (ii) −*x*+1, *y*, −*z*+1/2; (iii) −*x*+1/2, −*y*+1/2, −*z*+1; (iv) *x*−1/2, *y*+1/2, *z*; (v) −*x*+1, −*y*+1, −*z*+1; (vi) *x*−1, *y*+1, *z*; (vii) *x*, *y*+1, *z*.
